# Osseointegration Properties of Titanium Implants Treated by Nonthermal Atmospheric-Pressure Nitrogen Plasma

**DOI:** 10.3390/ijms232315420

**Published:** 2022-12-06

**Authors:** Sifan Yan, Satoshi Komasa, Akinori Agariguchi, Giuseppe Pezzotti, Joji Okazaki, Kenji Maekawa

**Affiliations:** 1 Department of Removable Prosthodontics and Occlusion, Osaka Dental University, 8-1, Kuzuhahanazono-cho, Hirakata-shi, Osaka 573-1121, Japan; 2 Ceramic Physics Laboratory, Kyoto Institute of Technology, Sakyo-ku, Matsugasaki, Kyoto 606-8126, Japan

**Keywords:** bone tissue engineering, titanium, nitrogen, nonthermal plasma, implant

## Abstract

Pure titanium is used in dental implants owing to its excellent biocompatibility and physical properties. However, the aging of the material during storage is detrimental to the long-term stability of the implant after implantation. Therefore, in this study, we attempted to improve the surface properties and circumvent the negative effects of material aging on titanium implants by using a portable handheld nonthermal plasma device capable of piezoelectric direct discharge to treat pure titanium discs with nitrogen gas. We evaluated the osteogenic properties of the treated samples by surface morphology and elemental analyses, as well as in vitro and in vivo experiments. The results showed that nonthermal atmospheric-pressure nitrogen plasma can improve the hydrophilicity of pure titanium without damaging its surface morphology while introducing nitrogen-containing functional groups, thereby promoting cell attachment, proliferation, and osseointegration to some extent. Therefore, nitrogen plasma treatment may be a promising method for the rapid surface treatment of titanium implants.

## 1. Introduction

A key task in ensuring successful implant restoration is improving implant stability for initial osteogenesis [1,2]. In the early stages of implantation, the interaction between the implant surface and various proteins, cells, etc., plays a significant role in initial osteogenic stability. A previous study showed that the long-term survival rates of titanium implants commonly used in clinical procedures are 89.23% and 82.94% after 10 and 16 years of follow-up, respectively [3]. The commercially available pure titanium and titanium alloys implants generally feature high biocompatibility, corrosion resistance, and good mechanical properties and strength [4,5,6,7]; however, owing to their inherent bio-inertness and ease of aging, their early osseointegration ability must be further improved to ensure the long-term success of titanium implants [8,9]. Thus, recent research has focused on improving the surface properties of these implants to enhance early osseointegration without adverse tissue reactions. Additionally, the modification of pure titanium surfaces is a popular research topic in the field of dental implants, as the surface properties of pure titanium play a decisive role in molecular interactions and cellular reactions [10,11]. The surface modification methods that have been widely studied include sandblasting [12], alkali treatment [13], acid etching [14], calcium phosphate deposition [15,16], oxidation [17], and ultraviolet irradiation [18], all of which can promote initial osteogenesis by increasing the bioactivity of the implant surface [7,19,20]. Among these technologies, plasma treatment is one of the most attractive research targets.

Plasma is a neutral ionized gas with high potential energy; it contains particles such as photons, electrons, positive and negative ions, atoms, free radicals, excited and non-excited molecules, etc. It can be obtained and applied to surface treatment using various techniques, including radio frequency glow discharge, electron cyclotron resonance discharge, corona discharge, atmospheric plasma processes, sputtering, chemical vapor deposition, plasma-assisted deposition, plasma implantation, plasma polymerization and grafting on polymer surfaces, and plasma spraying [21]. When the technical parameters are carefully controlled, plasma treatment can also be used to modulate surface chemical properties according to the specific application requirements of the material [22]. Nonthermal plasma can break C–C and C–H bonds on the implant surface and generate hydrophilic OH bonds and reactive oxygen and nitrogen species (RONS) to reduce carbon contamination on the implant surface, impart hydrophilicity, and increase the surface energy, resulting in a surface conducive to osseointegration [23,24,25]. Moreover, some reports have shown that increased hydrophilicity can inhibit bacterial adhesion [26]. Thus, nonthermal plasma-treated implant material surfaces may exhibit accelerated initial osseointegration by limiting bacterial adhesion in the early stages of implantation, which would reduce excessive inflammatory responses. 

Plasma treatment utilizing piezoelectric cold plasma generators to generate piezoelectric direct discharge (PDD) requires an operating power range of only 3–10 W; thus, it is well suited for compact benchtop or handheld atmospheric-pressure plasma applications [27]. The simplicity, convenience, low temperature, safety, and efficiency of such devices are also well suited for application prior to clinical implantation procedures. The handheld PDD air-based atmospheric-pressure plasma treatment of pure titanium surfaces has been shown to be effective in enhancing hydrophilicity and promoting the attachment of rat bone marrow mesenchymal stem cells (rBMMSCs) without changing the surface morphology of the metal [28]. Nitrogen plasma-treated surfaces have been suggested to provide a favorable environment for the osteogenic differentiation of osteoblasts [29,30,31,32]. A variety of nitrogen radicals, such as N_2_, N_2_ (excited), and N, contained within the nitrogen plasma can participate in chemical reactions on the material surface, forming different bio-polymeric bonds and improving surface wettability [33]. The direct treatment of MC3T3-E1 cells with low-temperature atmospheric-pressure nitrogen plasma does not affect the cell morphology, promotes the proliferation of preosteoblasts, and enhances osteogenic differentiation [34]. Nevertheless, the effect of treating pure titanium metal with nonthermal atmospheric-pressure nitrogen plasma on bone formation remains unknown. Therefore, in this study, we used nonthermal atmospheric-pressure nitrogen plasma to treat the surface of pure titanium discs, expecting to observe changes in the chemical composition of the specimen surface without changes in its morphology. Surface analysis, in vitro experiments using rBMMSCs, and in vivo experiments using a rat femur model were then conducted to investigate the effect of nonthermal atmospheric-pressure nitrogen plasma on osseointegration on pure titanium metal.

## 2. Results

### 2.1. Microstructure of the Materials

#### 2.1.1. Surface Morphology

**Figure 1:**
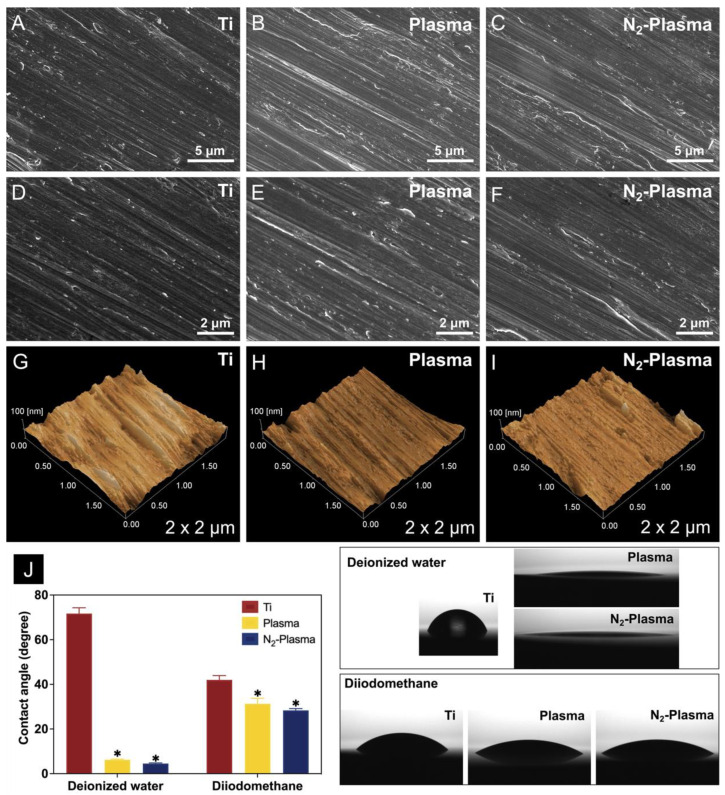
SEM analysis of the (**A**,**D**) Ti, (**B**,**E**) Plasma, and (**C**,**F**) N_2_-Plasma discs. Scanning probe micrographs of the (**G**) Ti, (**H**) Plasma, and (**I**) N_2_-Plasma surfaces. (**J**) Contact angles of the Ti, Plasma, and N_2_-Plasma discs. * *p* < 0.05 compared with Ti.

The surface characteristics of pure titanium discs without (Ti) and with nonthermal atmospheric-pressure plasma (Plasma) or nonthermal atmospheric-pressure nitrogen plasma (N_2_-Plasma) treatment were analyzed by scanning electron microscopy (SEM; S-4800; Hitachi, Tokyo, Japan). Images of the surface morphology of the different samples at different magnifications are shown in Figure 1A–F. Under 5000× and 10,000× magnification, the surfaces of the Plasma and N_2_-Plasma samples were clearly similar to those of the Ti samples, and both treated samples showed only grinding marks. 

Figure 1G–I shows the results of scanning probe microscopy (SPM; SPM-9600; Shimadzu). The surface morphological features of all samples were uniform and similar, and no significant difference in mean surface roughness (Ra) among the samples could be observed (Table 1). 

#### 2.1.2. Surface Wettability and Surface Energy 

The results of hydrophilic analysis in Figure 1J showed that the contact angles of deionized water and diiodomethane on the Ti surface were 71.7° and 41.7°, respectively; thus, the sample shows significant hydrophobicity. By contrast, the surfaces of the Plasma and N_2_-Plasma samples exhibited superhydrophilicity, as evidenced by contact angles of only 6.2° and 4.6°, respectively, for deionized water and 30.9° and 28.5°, respectively, for diiodomethane. Furthermore, the amount of polar surface components increased substantially for both Plasma and N_2_-Plasma samples, which showed higher surface energies overall (Table 2). No significant difference in surface energy between the two plasma treatment groups was observed. 

#### 2.1.3. Composition Analysis

Analysis of the surface elemental states of the samples was conducted using X-ray photoelectron spectroscopy (XPS; ESCA-5600; ULVAC-PHI, Kanagawa, Japan). 

**Figure 2:**
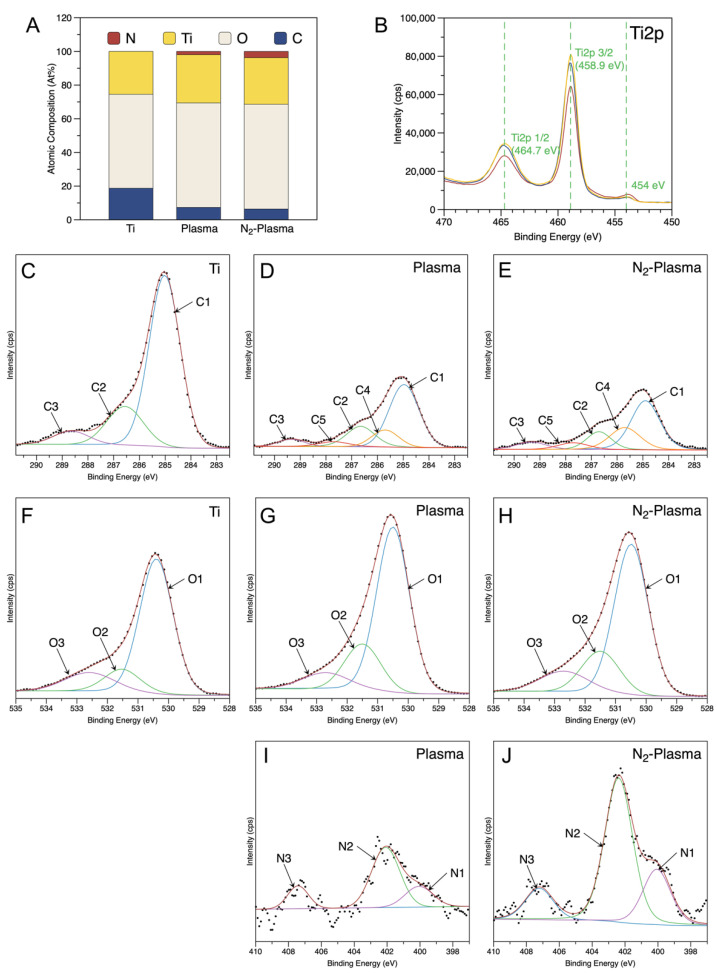
(**A**) Atomic percentage of each element on the sample surface. (**B**) High-resolution spectrum of Ti2p. Deconvoluted (**C**–**E**) C1s and (**F**–**H**) O1s XPS profiles of the Ti, Plasma, and N_2_-Plasma discs. Deconvoluted (**I**,**J**) N1s XPS profiles of Plasma, and N_2_-Plasma discs.

Figure 2A shows the surface elemental composition of all the samples. It was revealed that the surface of pure titanium discs consisted of titanium (25.44%), oxygen (55.84%), and carbon (18.72%). Similar to the results of previous studies, pure titanium discs showed carbon surface contamination [35]. After plasma and N_2_-plasma treatment, the content of carbon remarkably significantly decreased to 7.33% and 6.39%, respectively, whereas the content of titanium and oxygen increased. In addition, small amounts of nitrogen (1.97% and 3.74%, respectively) were found to be incorporated into the surfaces of plasma and N_2_-plasma-treated samples. 

Figure 2B shows Ti2p3/2 and Ti2p1/2 at 458.9 eV and 464.7 eV, respectively, on all the samples, indicating that titanium on the sample surface is mostly present in the Ti^4+^ valence state (TiO_2_). Additionally, a small peak was detected at 454 eV, corresponding to metallic titanium [36]. 

Figure 2C–E depicts the fitted C1s spectra. The spectra of the Ti sample show the C–C/C–H peak at 284.9 eV (C1), the C–O peak at 286.7 eV (C2), and the O=C–O peak at 288.6 eV (C3) (Figure 2C), indicating that the organic contamination on the pure titanium surface is mainly in the form of C–C/C–H bonds (74.17%) (Table 3). After plasma and N_2_-Plasma treatment, the relative C–C/C–H content was significantly reduced (56.31% and 47.55%), and new peaks at 285.7 eV and 287.7 eV emerged, corresponding to C–N (C4) and C=O/N–C=O (C5), respectively [37]. Compared with that in the Plasma sample, the peak area of the region reflecting nitrogen-related functional groups was higher in the N_2_-Plasma samples (Table 3). Furthermore, due to the increased introduction of nitrogen groups, the proportion of C–C/C–H on the surface of N_2_-Plasma samples is lower than on Plasma samples. Moreover, the binding energy of O=C–O peak on the treated samples was evaluated to 289.3 eV, possibly due to an increase in dicarboxylate (CO_2_^2-^) content as a result of the oxidation reaction during plasma and N_2_-plasma treatment. The increase in this kind of polar group may also improve the performance of cells [37]. 

Three oxygen atom states were revealed after the deconvolution of the O1s peak (Figure 2F–H) of all the samples [38,39]. The O1 peak corresponded to O^2−^ in the TiO_2_ lattice structure was located at 530.3 eV, the O2 peak corresponded to –OH groups was positioned at 531.4 eV, and NO_x_ or H_2_O (O3) groups at 532.7 eV [40]. The peak area ratio of the region representing –OH groups (O2) significantly increased in the Plasma and N_2_-Plasma specimens, respectively, compared with that in the control group, indicating enhanced surface hydroxylation (Figure 2F–H, Table 3). 

N1s signals were also detected on Plasma and N_2_-Plasma samples (Figure 2I,J). The peak at 400 eV was attributed to C–N bond [41], and the relative content on N_2_-Plasma samples was slightly more than on Plasma samples. The gas-phase reactive nitrogen species (RNS) such as NO produced during plasma and N_2_-Plasma treatment was absorbed on the sample surface and showed a peak near 402.3 eV on the N1s spectrum [40]. At the binding energy of 407.3 eV, the peak was corresponding to NO_x_ species (nitrates) [42].

### 2.2. Bioactivity Evaluation

#### 2.2.1. Cell Adhesion, Proliferation, and Morphology

**Figure 3:**
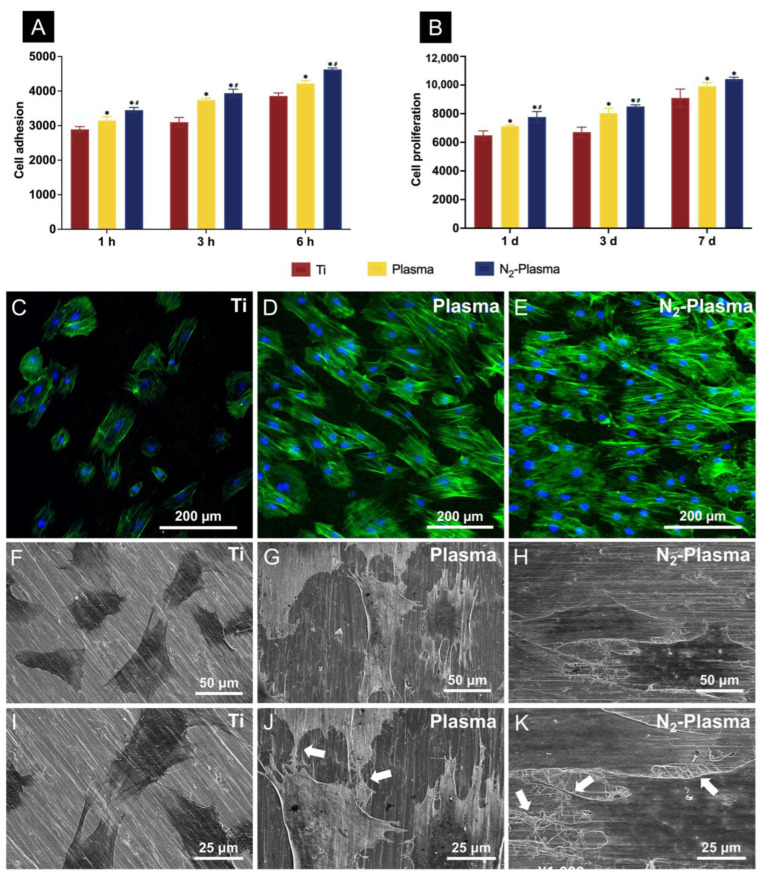
(**A**) Cell adhesion and (**B**) proliferation of rBMMSCs attached to the Ti, Plasma, and N_2_-Plasma discs. Morphological analysis of rat bone marrow mesenchymal stem cells (rBMMSCs) attached to the (**C**) Ti, (**D**) Plasma, and (**E**) N_2_-Plasma discs after 24 h of culture. Actin filaments (green) were labeled with Alexa Fluor 488 phalloidin, and nuclei (blue) were stained with 4′,6-diamidino-2-phenylindole. SEM analysis of the morphology of rBMMSCs attached to the (**F**,**I**) Ti, (**G**,**J**) Plasma, and (**H**,**K**) N_2_-Plasma discs after 24 h of culture. Images obtained at a lower magnification (**F**–**H**) show the morphology of single cells, whereas images obtained at a higher magnification (**I**–**K**) show the detailed interactions between cells on different samples (white arrows indicate connections between cells). * *p* < 0.05 compared with Ti; ^#^ *p* < 0.05 compared with Plasma.

The cell attachment results showed that, among the groups, the N_2_-plasma-treated surfaces had the most number of adherent cells at 1, 3, and 6 h after cell seeding; moreover, the Plasma group showed more adherent cells than the Ti group (Figure 3A). The results of cell proliferation tests conducted over 1, 3, and 7 d also showed the highest number of adherent cells on the N_2_-Plasma samples (Figure 3B). In addition, the morphological fluorescence staining results obtained 24 h after cell seeding indicated the greatest numbers of adherent cells on the Plasma and N_2_-Plasma samples; between these samples, more cells adhered on the N_2_-plasma-treated surface. Compared with those adhered to the Ti samples, the cells adhered on the surface of the two treated samples showed better stretching (Figure 3C–E). SEM revealed that the cells on the surface of the Plasma and N_2_-Plasma samples were more fully extended, closely adhered to the material surface, and connected to adjacent cells via extended filamentous pseudopods compared with those on Ti samples surface (Figure 3F–K). 

#### 2.2.2. Determination of Intracellular Reactive Oxygen Species (ROS)

**Figure 4:**
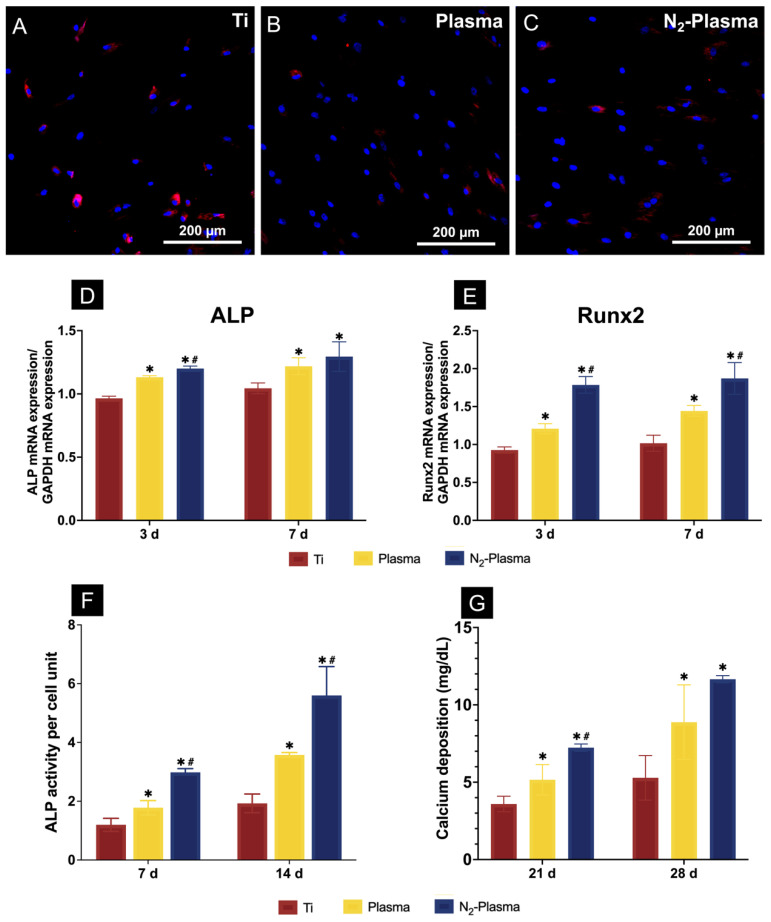
Reactive oxygen species generation of rat bone marrow mesenchymal stem cells on (**A**) Ti, (**B**) Plasma, and (**C**) N_2_-Plasma as determined by fluorescence microscopy. (**D**,**E**) Quantitative real-time PCR analysis of osteogenesis-related gene expression on Ti, Plasma, and N_2_-Plasma. (**F**) Alkaline phosphatase (ALP) activity on Ti, Plasma, and N_2_-Plasma. (**G**) Calcium deposition on Ti, Plasma, and N_2_-Plasma. * *p* < 0.05 compared with Ti; ^#^ *p* < 0.05 compared with Plasma.

The generation of intracellular ROS was detected by fluorescent staining after 24 h of incubation (Figure 4A–C). Cells on the surfaces of the Plasma and N_2_-Plasma samples showed lower levels of ROS production compared with those on the Ti samples. 

#### 2.2.3. Evaluation of Osteogenic Activity

The expression levels of osteogenesis-related genes, including alkaline phosphatase (ALP) and runt-related transcription factor 2 (Runx2), were assessed by TaqMan quantitative real-time polymerase chain reaction (PCR). ALP is considered one of the most reliable markers for early osteogenic differentiation [43,44]. Additionally, Runx2, a transcription factor belonging to the runt homology domain family, is required for osteoblast differentiation [45]. Compared with those on the Ti and Plasma samples, the cells grown on the N_2_-Plasma samples showed higher levels of ALP and Runx2 mRNA after 3 and 7 d of culture (Figure 4D,E). 

Figure 4F shows the quantitative results of ALP activity. The relative ALP activity of cells on both the Plasma and N_2_-Plasma samples was significantly higher than that of cells on the Ti sample. Between the plasma-treated samples, the N_2_-Plasma samples showed higher ALP activity. 

Calcium deposition, a late marker for extracellular matrix mineralization, in each group was quantified after differentiation for 21 and 28 d. The N_2_-Plasma samples showed the highest levels of calcium deposition at 21 and 28 d. Moreover, calcium deposition was significantly higher on the Plasma samples than on the Ti samples, as shown in Figure 4G. 

**Figure 5:**
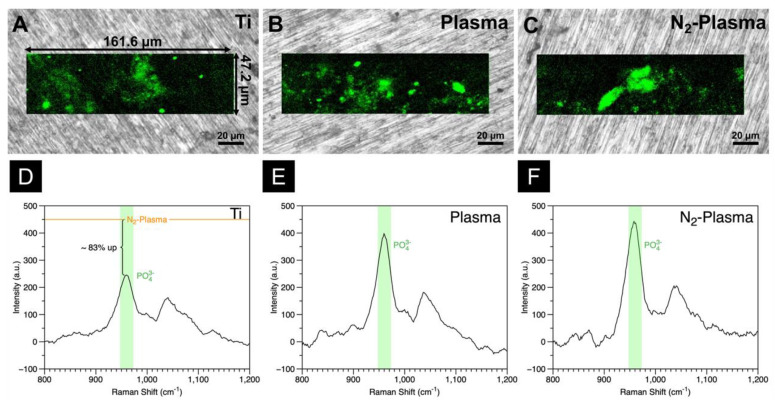
In situ Raman analyses of (**A**) Ti, (**B**) Plasma, and (**C**) N_2_-Plasma. Hydroxyapatite (HA) is displayed in green. Raman spectra of the PO_4_^3−^ stretching region, representing HA, of (**D**) Ti, (**E**) Plasma, and (**F**) N_2_-Plasma samples.

The extent of hydroxyapatite (HA) formation on the samples after cultivation for 21 d was quantitatively visualized via in situ Raman mapping, shown in Figure 5A–F. The peak at ~958 cm^−1^ reflects the symmetric stretching band of PO_4_^3−^. The intensity of the HA O–P–O band, the strongest Raman emission of inorganic molecules in bone apatite, is indicated by the green region in the Raman maps [46,47]. The larger area of the green region in the Raman map of the N_2_-Plasma sample confirmed the higher amount of HA on its surface. This finding contrasts the small spot-like green features on the Raman maps of the Ti and Plasma samples. Comparison of the Raman intensities of the O–P–O stretching bands of the samples shows that the apatite content in the N_2_-Plasma sample is 83% higher than that in the Ti sample and slightly higher than that in the Plasma sample. 

#### 2.2.4. In Vivo Evaluation of Bone Formation around Implants

**Figure 6:**
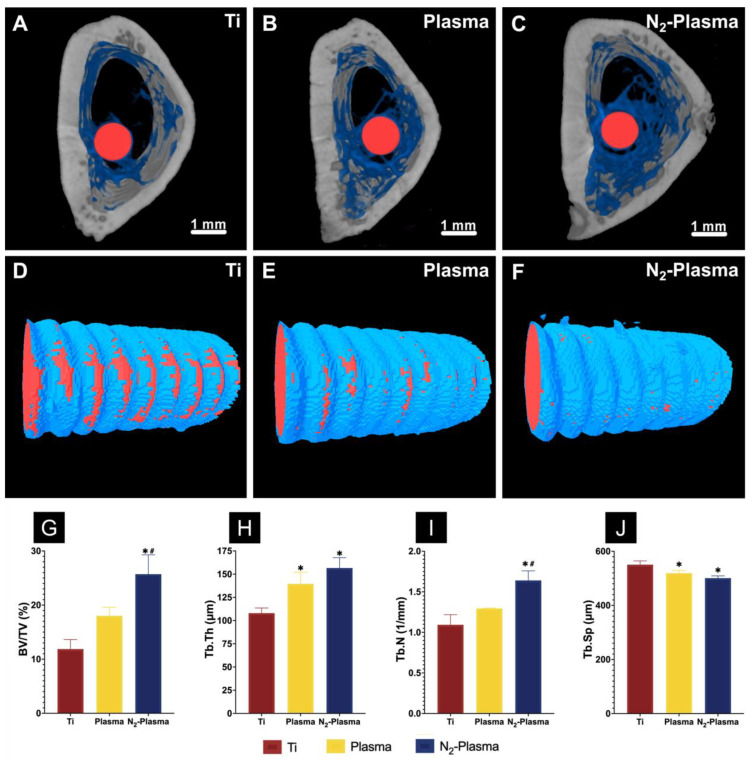
Reconstructed three-dimensional microcomputed tomography transverse slices of rat femurs containing (**A**) Ti, (**B**) Plasma, and (**C**) N_2_-Plasma implants after 8 weeks (red, implants; blue, cancellous bone; gray, cortical bone). Reconstructed three-dimensional micro-CT images of bone tissues around the (**D**) Ti, (**E**) Plasma, (**F**) N_2_-Plasma implants (red, implants; blue, new bone). (**G**) Bone volume to total volume ratio (BV/TV), (**H**) trabecular thickness (Tb. Th), (**I**) trabecular number (Tb. N), and (**J**) trabecular separation (Tb. Sp) around the Ti, Plasma, and N_2_-Plasma implants after 8 weeks. * *p* < 0.05 compared with Ti; ^#^ *p* < 0.05 compared with Plasma.

A rat femur model was selected to assess osteogenic activity around the implants in each group. Reconstructed three-dimensional microcomputed tomography (micro-CT) images of the implants are shown in Figure 6A–F. More trabecular microstructures could be observed around the Plasma and N_2_-Plasma implant surfaces than around the Ti surface after 8 weeks. Compared with the Ti group, new bone formation around the implants was higher in Plasma and N_2_-Plasma groups in Figure 6D–F. Additionally, the Plasma and N_2_-Plasma samples showed higher bone volume to total volume (BV/TV), mean trabecular number (Tb. N), and mean trabecular thickness (Tb. Th) than the Ti samples; between the plasma-treated samples, N_2_-Plasma showed higher values. The mean trabecular separation (Tb. Sp) was highest in the Ti implants (Figure 6G–J).

**Figure 7:**
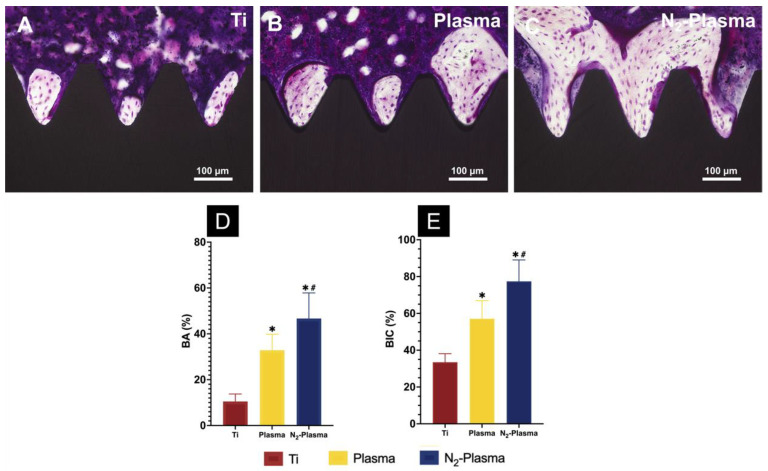
Histological sections of bone tissues around the (**A**) Ti, (**B**) Plasma, and (**C**) N_2_-Plasma implants. (**D**) Bone area ratio (BA) and (**E**) bone-to-implant contact (BIC) around the Ti, Plasma, and N_2_-Plasma implants. * *p* < 0.05 compared with Ti; ^#^ *p*< 0.05 compared with Plasma.

Images of the longitudinal sections of the implants and surrounding bone tissues are presented in Figure 7. In general, compact adherent new bone tissue was visible on the implant surfaces. However, more new bone cells were observed around the surfaces of the Plasma and N_2_-Plasma implants than around the surface of the Ti implant, as shown in Figure 7A–C. Histomorphometric analysis showed that the bone area ratio (BA) and bone-to-implant contact (BIC) were highest around the N_2_-Plasma implants, moderate around the Plasma implants, and lowest in the Ti implants (Figure 7D,E).

## 3. Discussion

The surface characteristics of an implant have a significant impact on its osseointegration and long-term stability after placement. Titanium, a biomaterial commonly used in clinical practice, often undergoes biodegradation from biologically active to bio-inert because of aging. Therefore, in this study, we used a portable handheld nonthermal atmospheric-pressure nitrogen plasma device for treating pure titanium implants to effectively improve their surface characteristics and facilitate early osseointegration while avoiding the negative effects of material aging.

Long-term storage inevitably causes organic contaminants from the atmosphere to deposit on the surface of the titanium implant, causing it to age and become hydrophobic [48,49]. Consistent with previous findings, the plasma and N_2_-plasma treatments in this study did not change the surface morphology and roughness of the titanium discs but effectively improved their hydrophilicity [28]. Such changes are associated with alterations in surface chemistry. After low-temperature plasma treatment, carbon contaminants are removed from the titanium surface, thereby exposing Ti^4+^ sites that can bind surrounding ·OH (hydroxyl radicals), which, in turn, enhances hydrophilicity [50]. In addition, TiO_2_ on the surface of titanium can generate oxygen vacancies by ultraviolet photocatalysis during plasma discharge and adsorb –OH to improve surface hydrophilicity [51]. The surfaces of titanium discs after plasma and N_2_-plasma treatment also exhibited higher surface energy. The high hydrophilicity and surface energy of the treated titanium surfaces effectively promote early cell attachment [52,53]. According to the cell adhesion results, more cells initially adhered to the surface of the plasma- and N_2_-plasma-treated samples than on the untreated samples, and cell extension was more extensive. Under the same roughness, materials with higher surface energy may show accelerated osseointegration at the early stages of implantation, which is beneficial to the long-term stability of implants [54]. 

It is well-known that there is a 3–7 nm thick TiO_2_ layer on the pure titanium surface [11]. The results of the elemental composition study showed that the carbon contamination on the surface of the titanium samples was significantly reduced and that their titanium and oxygen contents were relatively elevated after the plasma and N_2_-plasma treatments. Reduction of the carbon percentage on the material surface could improve protein and cell adhesion and promote osteoblast differentiation [55,56]. The O1s results showed that the content of –OH groups on the samples of both treatment groups increased substantially compared with that in the pure-titanium group. High-energy plasma breaks C–C/C–H/C–O bonds and forms free radicals, which subsequently generate various oxygen-containing functional groups, such as –OH, –COO, –CO_2_^2−^, etc., through a series of oxidation reactions [57,58]. –OH groups play a very important role in the early stages of cell adhesion as these groups are heavily depleted owing to their interaction with the titanium surface [59]. Therefore, we speculate that both the plasma and N_2_-plasma treatments effectively increase the content of –OH groups on the titanium surface, thus providing favorable conditions for cell attachment. We also observed that C–N and O=C–N functional groups were newly generated on the treated titanium surfaces, with higher contents found on the N_2_-plasma-treated samples than on the plasma-treated ones. We hypothesize that these nitrogen-containing functional groups are generated by chemical reactions between the radicals formed by the high-energy plasma bombardment of C–C/C–H bonds and nitrogen radicals. Extracellular fibronectin has been suggested to be closely related to cell adhesion. The sequence of Arg-Gly-Asp (RGD), the major integrin binding domain in fibronectin, is sensitive to nitrogen-containing functional groups and can regulate the signaling pathway mediated by integrins, thereby promoting cell adhesion, proliferation, and differentiation [60]. Positively charged nitrogen-containing functional groups also interact with negatively charged osteoblasts on the titanium surface, rendering it favorable for cell attachment and, thus, enhancing cell proliferation and osteogenic differentiation [61]. Nitrogen-containing groups may play a more prominent role in cell attachment than oxygen-containing groups [62]. Moreover, during the plasma treatment, gas-phase RNS such as NO was produced and absorbed into the samples, and it has been reported that moderate concentrations of NO may promote maturation and mineralization of the osteoblast extracellular matrix [63]. We believe that these mechanisms could explain the higher level of induction of hard tissue differentiation exhibited by the N_2_-plasma-treated group compared with that demonstrated by other groups in the in vitro experiments.

The cells on the N_2_-plasma-treated samples displayed higher adhesion, extension, and proliferation abilities at the initial stage of treatment, and neither plasma nor N_2_-plasma treatments caused significant damage to the cells or promoted apoptosis. The ROS staining results revealed lower intracellular ROS production on the samples treated with plasma and N_2_-plasma compared with pure titanium. On the one hand, intracellular ROS and nitrogen groups act as signaling molecules of oxidative stress to control the inflammatory response of the body; on the other hand, excessive ROS accumulation can damage cellular proteins, such as DNA, and lead to cell apoptosis [64,65]. It has been shown that lower levels of ROS are particularly important for the differentiation of stem cells [66]. The lower levels of ROS in the treated samples also showed a positive promotion of cell growth, proliferation, and bone-forming differentiation compared to the titanium surface in the present experiment. It has also been demonstrated that neutral ROS generated during plasma treatment, such as NO, H_2_O_2_, or OH may affect the release of fibroblast growth factor-2 (FGF-2) [67]. FGF-2 can enhance the proliferation of osteoblasts and BMMSCs, thereby regulating their osteogenic function [68,69,70]. This may also be related to the results of the present experiment. In the future, more focus can be placed on studying the effects of plasma-generated ROS on osteogenic function. 

The real-time PCR results indicated that the expression of osteogenic-related genes ALP and Runx2 was highest in the N_2_-plasma-treated samples. Furthermore, the ALP and calcium deposition results indicated that the cells on the N_2_-plasma-treated samples showed a high level of differentiation ability for osteogenesis. Raman cell imaging also revealed the highest amount of HA deposition on the N_2_-plasma surface. These results confirm that N_2_-plasma treatment has a positive effect on initial osteogenic differentiation in vitro. In vivo experimental results also revealed the most extensive new bone formation on the surface of the N_2_-plasma-treated implants. We thus speculate that N_2_-plasma treatment can improve the osseointegration of titanium implants, which has a positive effect on their long-term stability. This effect may be related to the strong correlation between the increased hydrophilicity and surface energy of the implant surface and changes in its chemical composition. 

The plasma and N_2_-plasma treatments produced a certain amount of gas-phase RONS, which has been shown to effectively promote bacterial death on material surfaces by inducing membrane changes and enzyme inhibition [71]. Nitrogen-containing functional groups can also inhibit bacterial attachment, thereby endowing implants with antibacterial properties [72,73]. Thus, N_2_-plasma treatment may also confer antimicrobial properties to implants and render them more suitable for use in the complex environment of dentistry; this supposition can be validated in future bacteria-related experiments. 

In summary, both N_2_-plasma and plasma treatments promoted the adhesion, proliferation, and osteogenic differentiation of rBMMSCs on the implants without altering the physical morphology of the pure titanium surface. The in vivo and in vitro results collectively indicated that N_2_-plasma leads to better osseointegration properties, which could be correlated with increases in the hydrophilicity and surface energy and modification of the surface chemistry of the pure titanium surface owing to the introduction of nitrogen-containing functional groups. On the other hand, both the reduction of organic contaminants and the introduction of nitrogen-containing functional groups after N_2_-plasma treatment occurred in the surface organic contamination layer of pure titanium implants; therefore, we assume that a similar activation effect might be obtained by performing the same treatment on titanium alloys. The portable handheld nonthermal plasma device used in this study is highly suitable for clinical dental implant use because of its low cost, convenience, and rapid effectiveness. Future experiments will be conducted to assess the potential antimicrobial properties of the proposed technique and simulate the complex environment of the oral cavity to optimize its clinical use.

## 4. Materials and Methods

### 4.1. Sample Preparation

Titanium discs (JIS Grade 2, Daido Steel, Osaka, Japan), measuring 15 mm in diameter and 1 mm in thickness, were chosen for this study. The discs were polished with SiC abrasive paper (Waterproof Paper^®^ Nos. 1000; Riken Corundum Co. Ltd., Saitama, Japan), cleaned with acetone, ethanol, and deionized water, each for 10 min, using an ultrasonic machine, and dried overnight at room temperature (23–25 °C). All of the samples were packed and then sterilized using dry heat at 160 °C for 3 h. 

### 4.2. Plasma Treatment

A nonthermal atmospheric-pressure handheld plasma device (Piezobrush^®^ PZ2, Relyon Plazma GmbH, Regensburg, Germany) utilizing PDD technology with a multigas-nozzle was used for sample treatment. The distance between the titanium discs and the jet exit was kept at 5 mm, and the treatment time was set to 30 s. The air plasma-treated (Plasma) discs were treated at room temperature (23–25 °C) with air-induced plasma at atmospheric pressure (0.1 MPa), and the nitrogen plasma-treated (N_2_-Plasma) discs were treated with N_2_-induced plasma (gas flow, 2 SLM) at atmospheric pressure (0.1 MPa). Untreated titanium (Ti) discs were tested as the control group, whereas the Plasma and N_2_-Plasma discs were tested as the experimental groups.

### 4.3. Surface Characterization

The surface morphology of the Ti, Plasma, and N_2_-Plasma samples was examined by SEM (S-4800; Hitachi, Tokyo, Japan) at a 5 kV accelerating voltage. 

Ra and surface topography in a range of 2 μm × 2 μm were assessed using a scanning probe microscope (SPM; SPM-9600; Shimadzu, Kyoto, Japan). 

One-microliter droplets of deionized water and diiodomethane were dropped on the surfaces of samples from each group using a surface measuring instrument (DropMaster DMs-401, Kyowa Interface Science Co., Ltd., Tokyo, Japan) at room temperature (23–25 °C) to assess their contact angle and surface energy. Images of the sample surfaces were captured, and the data were analyzed using an interface measurement and analysis system (FAMAS, Kyowa Interface Science Co., Ltd., Tokyo, Japan) to evaluate the surface energy of all samples via the Owens–Wendt–Rabel–Kaelble method [74]. 

XPS (ESCA-5600; ULVAC-PHI, Kanagawa, Japan) equipped with a monochromatic X-ray source (Al Kα) was used to examine the surface-specific chemical states of the samples. Then the elements were examined using the Shirley background and the relative sensitivity coefficients given by the instrument manufacturer using Multipak software (Multipak v9.6.1; ULVAC-PHI, Kanagawa, Japan). The C1s spectrum was used to calibrate the energy scale (285.0 eV). 

### 4.4. In Vitro Experiments

#### 4.4.1. Cell Culture

Rat BMMSCs (rBMMSCs) obtained from the femurs of eight-week-old Sprague-Dawley rats (Shimizu Laboratory Supplies Co., Kyoto, Japan) were used in this study and cultured in Eagle’s minimum essential medium (E-MEM) containing 10% fetal bovine serum (FBS) and an antibiotic–antimycotic solution (all from Nacalai Tesque Inc., Kyoto, Japan) in 75 cm^2^ flasks according to a previously reported method [13]. Third-generation cells were used in the in vitro experiments. The cells were digested in a solution containing 0.5 g/L trypsin and 0.53 mmol/L EDTA (Nacalai Tesque Inc., Kyoto, Japan), centrifuged, resuspended, and added to disc samples (Ti, Plasma, and N_2_-Plasma) placed in a 24-well plate at a density of 4 × 10^4^ cells/well. The cell culture medium was changed every 3 d. This study was performed in accordance with the Guidelines for Animal Experimentation at Osaka Dental University (Approval No. 22-08002).

#### 4.4.2. Cell Adhesion and Proliferation

Cell adhesion after 1, 3, and 6 h and cell proliferation after 1, 3, and 7 d were evaluated using the CellTiter-Blue^®^ Cell Viability Assay (Promega Corporation, Madison, WI, USA) according to the manufacturer’s protocol. After incubation for 1 h, 3 h, 6 h, 1 d, 3 d, and 7 d, the samples were washed twice with PBS and added with 300 μL of diluted Cell Titer-Blue^®^ Reagent (50 μL Cell Titer-Blue^®^ Reagent in 250 μL of PBS). After culturing for 1 h at 37 °C with 5% CO_2_, 100 μL of the reagent in each well was added to a 96-well plate. The fluorescence of the solutions was analyzed at 560/590 nm using a microplate reader (SpectraMax M5; Molecular Devices, San Jose, CA, USA). 

#### 4.4.3. Cell Morphology

The cell morphology of all samples after 24 h of culture was observed by fluorescence staining and SEM.

For fluorescence staining, samples that had been incubated for 24 h were washed thrice with PBS, fixed by adding 1 mL of 4% paraformaldehyde (PFA) solution, and cultured for 20 min at room temperature (23–25 °C). The samples were subsequently washed thrice with PBS. The cells were permeabilized by adding 0.2% (*v*/*v*) Triton X-100 to the samples. After shaking for 30 s and culture for 30 min, the samples were treated with Blocking One reagent (Nacalai Tesque Inc., Kyoto, Japan) for 30 min at room temperature (23–25 °C) and stained with Alexa Fluor 488 phalloidin and 4′,6-diamidino-2-phenylindole (DAPI) at 37 °C in the dark for 1 h. The stained samples were washed four times with PBS. F-actin and cell nuclei were observed using a confocal laser-scanning microscope (LSM700; Carl Zeiss AG, Wetzlar, Germany). 

For SEM analysis, cells adhered on the samples after 24 h of culture were washed thrice with PBS (Gibco^TM^, Thermo Fisher Life Technologies Ltd., Tokyo, Japan) and fixed with 4% PFA for 2 h. The cells were then sequentially dehydrated with a range of ethanol concentrations (50%, 60%, 70%, 80%, 90%, 99%, and 99.5%) for 10 min each. The samples were dried in a critical point desiccator (HCP-1; Hitachi, Tokyo, Japan), coated with osmium using an ion sputterer (HPC-20; Vacuum Device, Ibaraki, Japan), and observed by SEM (S-4800; Hitachi, Tokyo, Japan).

#### 4.4.4. ROS Production

Intracellular ROS production was detected by fluorescent staining. After 24 h of cell seeding, all samples were washed thrice with PBS, incubated with 1 mL of antibiotic-free medium containing 5 μM CellROX^®^ oxidative stress reagent (C10422, Thermo Fisher Life Technologies Ltd., Tokyo, Japan) and DAPI at 37 °C for 30 min, and then fixed with 4% PFA for 15 min. Imaging was performed with an LSM700 confocal laser scanning microscope (LSM700; Carl Zeiss AG, Wetzlar, Germany).

#### 4.4.5. Osteogenesis-Related Gene Expression 

A TaqMan real-time PCR assay (Life Technologies, Carlsbad, CA, USA) was performed to assess the expression levels of osteogenesis-related genes, as previously described [13]. After culture for 3 or 7 d on the samples, the total RNA of rBMMSCs was harvested using an RNeasy Mini Kit (Qiagen, Venlo, The Netherlands). The same amount (10 μL) of each RNA sample was reverse-transcribed into cDNA using a PrimeScript RT kit (TaKaRa Bio, Shiga, Japan). The StepOneTM Plus real-time PCR System (Life Technologies, Carlsbad, CA, USA) was selected to quantitatively detect the expression levels of ALP and Runx2 after 3 and 7 d of culture. The expression rate of reactive genes in each group was normalized to that of the housekeeping gene, glyceraldehyde 3-phosphate dehydrogenase (GAPDH), and calculated using the ΔΔCt method.

#### 4.4.6. ALP Activity

α-MEM (Nacalai Tesque Inc., Kyoto, Japan), a differentiation-inducing medium, containing 10% FBS, antibiotic–antimycotic mix, and the osteogenic supplements 10 mM β-glycerophosphate (Wako Pure Chemical Industries Ltd., Osaka, Japan), and 10 nM dexamethasone (Nacalai Tesque Inc., Kyoto, Japan) was used instead after the cells were cultured for 1 week in E-MEM containing 10% FBS and an antibiotic–antimycotic solution (all from Nacalai Tesque Inc., Kyoto, Japan). The medium was changed every 3 d. The samples were cultured for 7 or 14 d, washed with PBS, and added with 300 μL of 0.2% Triton X-100 to achieve cell lysis. The lysates were transferred to microcentrifuge tubes. ALP pNPP Liquid Substrate from an enzyme-linked immunosorbent assay kit (Sigma-Aldrich, St Louis, MO, USA) was used to determine ALP activity following the manufacturer’s protocol. Exactly 50 μL of 3 M NaOH was added to 200 μL of the reaction substrate to terminate the reaction. *p*-Nitrophenol production was determined by detecting the optical density of the solution at 405 nm using a 96-well microplate reader (SpectraMax^®^ M5; Molecular Devices, San Jose, CA, USA). DNA content was determined using a PicoGreen dsDNA Assay Kit (Thermo Fisher Scientific, Waltham, MA, USA) according to the manufacturer’s protocol. ALP content was normalized to the DNA content of the corresponding cell lysates.

#### 4.4.7. Calcium Deposition in the Extracellular Matrix

After culture with differentiation-inducing medium for 21 or 28 d, as described in Section 4.4.6, the calcium deposits in the extracellular matrix on the samples were dissolved with 10% formic acid and collected. A Calcium E-Test Kit (Wako Pure Chemical Industries Ltd., Osaka, Japan) was used to quantify the amount of calcium in these deposits. Exactly 50 μL of the collected medium was added with 1 mL of calcium emission test reagent and 2 mL of kit buffer. The reaction products were then detected using a 96-well microplate reader (SpectraMax^®^ M5; Molecular Devices, San Jose, CA, USA) at 610 nm. The calcium concentration was calculated from the absorbance of the relative standard curve.

#### 4.4.8. Raman Imaging 

After culture with a differentiation-inducing medium for 21 d, the samples were treated with 1 mL of 4% PFA solution and cultured for 2 h at room temperature (23–25 °C). Then, the HA produced during the osteogenic mineralization of the cells on the samples was detected and imaged by a confocal laser Raman microscope (RAMAN-touch, Nanophoton Co., Ltd., Osaka, Japan) equipped with a 532 nm wavelength laser source. The relative intensity of the HA principal band, located at 958 cm^−1^, is related to O–P–O stretching in the PO_4_^3−^ tetrahedron [46,47]. 

### 4.5. In Vivo Experiments

#### 4.5.1. Animal Model and Surgical Procedures

The animal experiment was performed according to the ethical principles of the National Animal Care Guidelines and approved by the Medical Ethics Committee of Osaka Dental University, Japan (Approval No. 22-08002). A total of 24 eight-week-old male Sprague-Dawley rats (Shimizu Laboratory Supplies Co., Kyoto, Japan), weighing 180–200 g each, were randomly divided into two groups. The surgical procedures used in this study have been previously described [75]. After general anesthesia and surgical cleaning, a 10 mm longitudinal incision was made along the medial side of the knee joint of the right hind leg. The patella and extensor mechanism was then dislocated to expose the distal femur. A 1.2 mm hole was drilled into the intercondylar notch using a dental burr with sterilized saline irrigation. Screws were implanted into the prepared channels, the knee joint was restored, and the incision was sutured. Gentamicin (1 mg/kg) and buprenorphine (0.05 mg/kg) were injected for 3 d after surgery to prevent postsurgical infection and decrease postoperative pain.

#### 4.5.2. Micro-CT

After 8 weeks, the rats were anesthetized and euthanized. Right femurs including the implants were placed in the saline solution immediately after dissection and scanned with a micro-CT system (SkyScan1275, Bruker, Billerica, MA, USA) operated at 90 kV and 40 μA with an aluminum filter. The BV/TV, Tb. N, Tb. Th, and Tb. Sp of the region of interest, defined as 2 mm below the highest point of the growth plate and extending 500 μm around each implant, were quantified to evaluate bone regeneration using morphometric software (CTAn; Bruker, Billerica, MA, USA). 

#### 4.5.3. Histology of Sequentially Labeled Sections

After micro-CT scanning, specimens harvested at 8 weeks were fixed in 70% ethanol solution for 3 d and then stained using the Villanueva method to assess bone generation. The histomorphometric characteristics of the sections were analyzed with a BZ-9000 digital cold illumination microscope (Keyence Co., Osaka, Japan). The BA and BIC around the implants were assessed using ImageJ software at a magnification of 200×.

### 4.6. Statistical Analysis

Surface analysis and in vitro experiments were conducted in triplicate. All quantitative results are expressed as means ± standard deviations. Data were analyzed using one-way analysis of variance and Bonferroni’s post hoc test. A *p* value of <0.05 was considered to be significant.

## 5. Conclusions

Nonthermal atmospheric-pressure nitrogen plasma treatment can effectively improve the hydrophilicity of pure titanium surfaces, increase their surface energy, reduce surface organic contamination, and introduce relatively more nitrogen-containing groups, thereby promoting cell adhesion and proliferation, enhancing initial osseointegration. The good osseointegration and potential antimicrobial properties of the fast, portable, and effective handheld nonthermal atmospheric-pressure nitrogen plasma treatment may render it an effective method for modifying the surface of clinical dental implants and circumventing material aging.

## Figures and Tables

**Table 1:** Surface roughness values of the Ti, Plasma, and N_2_-Plasma samples. Data shown are the means ± SD (*n* = 3).

	Ti	Plasma	N_2_-Plasma
Ra (nm)	9.96 ± 1.94	9.64 ± 3.36	9.84 ± 1.57

**Table 2:** Surface energies of the Ti, Plasma, and N_2_-Plasma samples.

	Ti	Plasma	N_2_-Plasma
Dispersive (mJ/m^2^)	32.7	28.3	29.2
Polar (mJ/m^2^)	9	44.7	44.1
Total energy (mJ/m^2^)	41.7	73	73.3

**Table 3:** Percentages of fitted peaks derived from the XPS results shown in Figure 2.

	Ti	Plasma	N_2_-Plasma
C1s peak Proportion (%)			
C1 (C–C/C–H)	74.17	56.31	47.55
C2 (C–O)	19.35	19.07	14.85
C3 (O–C=O)	6.48	5.68	7.62
C4 (C–N)	---	14.03	22.95
C5 (C=O/N–C=O)	---	4.9	7.03
O1s peak Proportion (%)			
O1 (Ti–O)	71.62	69.66	67.18
O2 (–OH)	13.9	20.49	20.08
O3 (NO_x_/H_2_O)	14.48	9.85	12.74
N1s peak Proportion (%)			
N1 (C-N)	---	21.02	23.97
N2 (NO_x_)	---	61.64	62.35
N3 (NO_x_)	---	17.35	13.69

## Data Availability

Not applicable.
